# Epidemiology and genetic diversity of invasive *Neisseria meningitidis* strains circulating in Portugal from 2003 to 2020

**DOI:** 10.1007/s10123-023-00463-w

**Published:** 2023-12-07

**Authors:** Célia Bettencourt, Alexandra Nunes, Paulo Nogueira, Sílvia Duarte, Catarina Silva, João Paulo Gomes, Maria João Simões

**Affiliations:** 1 National Reference Laboratory for Neisseria Meningitidis, Department of Infectious Diseases, National Institute of Health Doutor Ricardo Jorge (INSA), Lisbon, Portugal; 2 Genomics and Bioinformatics Unit, Department of Infectious Diseases, National Institute of Health Doutor Ricardo Jorge (INSA), Lisbon, Portugal; 3 Veterinary and Animal Research Centre (CECAV), Faculty of Veterinary Medicine, Lusófona University, Lisbon, Portugal; 4 Escola Nacional de Saúde Pública, NOVA University of Lisbon, Lisbon, Portugal; 5 Innovation and Technology Unit, Department of Human Genetics, National Institute of Health Doutor Ricardo Jorge (INSA), Lisbon, Portugal

**Keywords:** *Neisseria meningitidis*, Epidemiology, Surveillance, Whole genome sequencing, Portugal

## Abstract

Invasive meningococcal disease (IMD) continues to be a public health problem due to its epidemic potential, affecting mostly children. We aimed to present a detailed description of the epidemiology of IMD in Portugal, including insights into the genetic diversity of *Neisseria meningitidis* strains. Epidemiological analysis included data from the Portuguese National Reference Laboratory of *Neisseria meningitidis* during 2003 to 2020. Since 2012, *N. meningitidis* isolates have also been assessed for their susceptibility to antibiotics and were characterized by whole genome sequencing. During 2003–2020, 1392 confirmed cases of IMD were analyzed. A decrease in the annual incidence rate was observed, ranging from 1.99 (2003) to 0.39 (2020), with an average case fatality rate of 7.1%. Serogroup B was the most frequent (69.7%), followed by serogroups C (9.7%), Y (5.7%), and W (2.6%). Genomic characterization of 329 isolates identified 20 clonal complexes (cc), with the most prevalent belonging to serogroup B cc41/44 (26.3%) and cc213 (16.3%). Isolates belonging to cc11 were predominantly from serogroups W (77.3%) and C (76.5%), whereas cc23 was dominant from serogroup Y (65.7%). Over the past 4 years (2017–2020), we observed an increasing trend of cases assigned to cc213, cc32, and cc11. Regarding antimicrobial susceptibility, all isolates were susceptible to ceftriaxone and 61.8% were penicillin-nonsusceptible, whereas 1.4% and 1.0% were resistant to ciprofloxacin and rifampicin. This is the first detailed study on the epidemiology and genomics of invasive *N. meningitidis* infections in Portugal, providing relevant data to public health policy makers for a more effective control of this disease.

**Supplementary Information:**

The online version contains supplementary material available at 10.1007/s10123-023-00463-w.

## Introduction

Invasive meningococcal disease (IMD) is caused by the gram-negative bacteria *Neisseria meningitidis*, a frequent commensal of the human nasopharynx that can occasionally lead to severe life-threatening illness. Meningitis and sepsis are the most frequent, affecting mostly children and young adults, but other less frequent syndromes, such as pneumonia, arthritis, epiglottitis, conjunctivitis, and pericarditis, may also occur (ECDC 2022a; WHO 2022; Tzeng and Stephens 2000; Rosenstein et al. 2001; Stephens and Apicella 2015).

*N. meningitidis* is classified into 12 serogroups based on the antigenic characteristics of its capsular polysaccharide. However, IMD is worldwide caused mainly by six serogroups, namely A, B, C, W, Y, and more recently X (Harrison et al. 2013). In developed countries, serogroups B, Y, and W account for the great majority of cases of IMD (ECDC 2022b; CDC 2022).

The incidence of IMD varies geographically and over time, mostly due to changes in the distribution of serogroups, mass gathering events, movements of population, and control policies (Chang et al. 2012; Kinlin et al. 2009; Muttalif et al. 2019). Since countries implemented meningococcal surveillance programs with different national coverage and accuracy, the real burden of the disease, as well as the genomic diversity of strains, remains unknown in vast geographic regions.

Invasive meningococcal disease is endemic in Europe and North America, occurring as sporadic cases with a low incidence rate (< 0.5 to 0.9 cases *per* 100,000 population) (Pelton 2016). The highest incidence of the disease is observed in the African meningitis belt, a sub-Saharan region that extends from Senegal to Ethiopia. Here, devastating epidemic waves periodically occur during the dry and windy season, with 10 to 1000 cases per 100,000 population (Pelton 2016; Sultan et al. 2005).

Vaccination is the most effective strategy for preventing meningococcal disease, and several vaccines against serogroups A, B, C, Y, and W are currently available (Dretler et al. 2018; Vuocolo et al. 2018). However, vaccine schemes do not cover all age groups and no vaccine is 100% effective. Nevertheless, the success of vaccine introduction in immunization programs has led to a shift in disease epidemiology with the decrease of IMD incidence in most countries (ECDC 2022b; CDC 2022; Lahra et al. 2022). In Portugal, the monovalent serogroup C (MenC) conjugate vaccine (MCC) was included in the National Immunization Programme in 2006, being recommended for children above 3 months of age as a 2 + 1 dose, with a catch-up campaign conducted in 2006 and 2007 targeting adolescents under the age of 19 (DGS 2005). This very successful strategy, reaching a vaccination coverage of 95%, resulted in a sharp decrease of cases due to MenC (Simões and Martins 2020). To prevent IMD caused by serogroup B (MenB), two vaccines are available in Portugal: the 4CMenB vaccine, accessible since April 2014 and included in the Portuguese routine immunization program in October 2020, being recommended for children above 2 months of age, and the bivalent rLP2086 vaccine, approved in 2017 for individuals older than 10 years (SPP 2018; DGS 2020).

With the purpose of improving the knowledge on the epidemiology of IMD, a surveillance system based on mandatory clinical and laboratory notifications was implemented in Portugal by the end of 2002. It is ruled by the General Directorate of Health (GDH) and the laboratory coordination is held by the National Reference Laboratory (NRL) for Neisseria meningitidis at the Portuguese National Institute of Health Doutor Ricardo Jorge (INSA) (DGS 2002). This system gathers clinical (clinical presentation and outcome) and laboratory data on case confirmation, as well as strains’ characterization (genotyping) from nationwide cases. Furthermore, since 2017, whole genome sequencing (WGS) has been routinely implemented at the NRL as a reference typing method for IMD surveillance according to the European Centre for Disease Prevention and Control (ECDC) guidelines (ECDC 2019).

This work presents a retrospective analysis of the epidemiology of IMD in Portugal from 2003 to 2020. It also analyzes the genetic diversity and population structure of the Portuguese invasive *N. meningitidis* strains during a 9-year period (from 2012 to 2020).

## Methods

### The Portuguese laboratory-based surveillance system of IMD

**Fig. 1:**
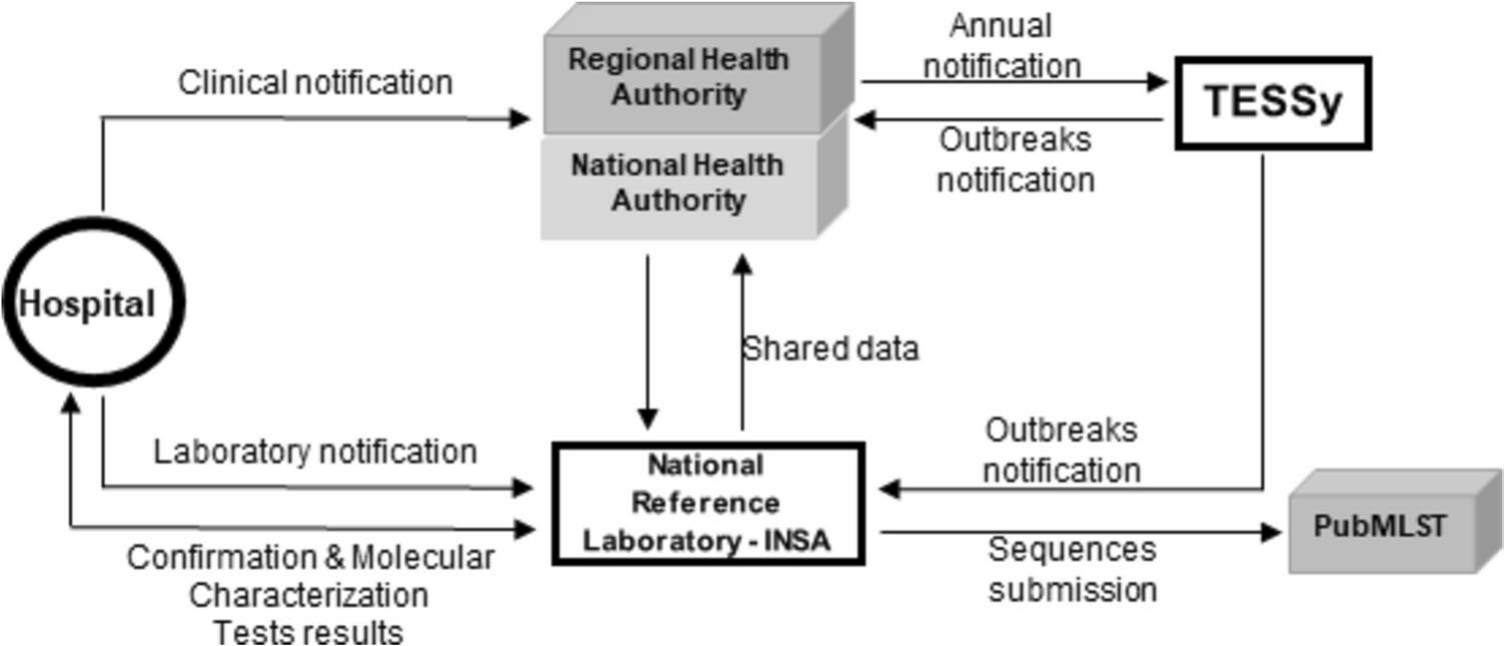
Diagram depicting the overall data flow of the IMD surveillance system in Portugal

In compliance with the GDH guidelines, clinicians must notify all cases of suspected IMD to the health authority, including demographic data, clinical presentation, and outcome. Since 2015, this is an online notification (SINAVE platform) simultaneously addressed to the local and national public health authorities. It is the responsibility of the local authority to carry out the epidemiological survey, in order to implement procedures for controlling and breaking down the disease transmission chain. Laboratory investigation of suspected cases is mandatory, as well as lab notification of confirmed IMD cases also through the SINAVE platform (https://sinave.min-saude.pt/SINAVE.MIN-SAUDE/login.html) (Fig. 1). Therefore, clinical samples from suspected cases of IMD and *N. meningitidis* isolates are sent by hospitals to the NRL, for confirmation, molecular characterization, and antibiotic susceptibility testing. Data from the NRL is sent back to the hospitals and to the GDH. Additionally, every year, Portuguese information is reported to the European Surveillance System (TESSy, ECDC).

This study included all cases with a date of onset from the 1st of January 2003 to the 31st of December 2020. Any discrepancies observed between the data presented in this document and the data in the TESSy reports or the general reports on mandatory communicable disease result from the comprehensive analysis of the national IMD database, which, on the other hand, gathers data from the different sources feeding the IMD surveillance system.

Demographic data (age, region of residence, date of onset, and outcome) was accessed from laboratory notification, in order to investigate a possible association with genomic traits.

### Bacterial isolates and serogroup identification

Isolates were cultured on sheep blood agar and incubated overnight at 37 °C under 5% CO2. DNA extraction and purification were performed using the automated NUCLISENS easyMAG system (bioMérieux, France), according to the manufacturer’s instructions. Meningococcal identification and group characterization (A, B, C, W, and Y) were performed by real-time PCR with specific primers and probes, according to the CDC protocols (WHO 2011).

### Antimicrobial susceptibility testing

This study included all viable invasive strains of *N. meningitidis* received in the NRL from 2012 (year from which the antibiotic susceptibility profile of all isolates received at the NLR was studied and characterized by WGS) to 2020. Antibiotic susceptibility testing was restricted to those used for treatment, namely penicillin and ceftriaxone, and prophylaxis, namely rifampicin and ciprofloxacin (Nadel 2016; Vygen et al. 2016). The antimicrobial susceptibility was expressed as the minimum inhibitory concentration (MIC), using the antibiotic gradient strip diffusion method (Etest, bioMérieux, France) on Mueller–Hinton agar (Thermo Scientific, USA), supplemented with 5% sheep blood. The breakpoints used for the classification of susceptible (S), susceptible, increased exposure (I) (to penicillin), and resistant (R) were those recommended by the European Committee for Antimicrobial Susceptibility Testing (EUCAST) guidelines (EUCAST 2020). In this study, we classified isolates as non-susceptible to penicillin, those with reduced susceptibility (susceptible, increased exposure), and/or fully resistant.

### Whole-genome sequencing (WGS) andde novoassembly

All invasive *N. meningitidis* isolates identified by the Portuguese NRL from 2012 to 2020 were characterized by WGS and sequences were submitted to the PubMLST database (https://pubmlst.org/organisms/neisseria-spp). For WGS, DNA samples were quantified with a Qubit Fluorometer using the DNA HS Assay Kit (ThermoFisher Scientific), while quality assessment was performed by agarose gel electrophoresis (0.8%). DNA was then subjected to dual-indexed Nextera XT Illumina library preparation (Illumina), before cluster generation and paired-end sequencing (2 × 150 bp or 2 × 250 bp) on a MiSeq, NextSeq 550, or NextSeq 2000 equipment (Illumina), available at INSA, according to the manufacturer’s instructions. Over the years, genome sequences were de novo assembled using INNUca v4.0.1, v4.0.5, or v4.2.0 (https://github.com/B-UMMI/INNUca), an integrative bioinformatics pipeline for read quality analysis and improvement, de novo assembly and polished (Llarena et al. 2018). For all isolates, in silico MLST and finetyping were performed using the meningotype v0.8.2-beta-01 platform (https://github.com/MDU-PHL/meningotype) (Kwong et al. 2010) and the PubMLST.org/neisseria database (http://pubmlst.org/neisseria/) (Jolley and Maiden 2010).

### Genetic diversity of the Portuguese invasive *N. meningitidis* isolates

The genetic relationship among all the Portuguese (PT) studied isolates from the period 2012–2020 was evaluated through a gene-by-gene analysis using the panel of 1422 loci that constitutes the newly improved MLST core-genome scheme (cgMLST) V2 for *N. meningitidis*, available on the PubMLST.org/neisseria database (Jolley et al. 2018). The genome comparator module was used to evaluate the genetic relatedness of all genomes and to generate a distance matrix of allelic differences. ReporTree software (Mixão et al. 2023) (https://github.com/insapathogenomics/ReporTree) was then applied to (i) perform a phylogenetic analysis based on the number of shared cgMLST alleles, with unique allelic profiles on a hierarchical single-linkage clustering criterion; (ii) identify potential genetic clusters (based on a generated MSTreeV2); and (iii) identify specific associations among variables of interest (serogroup, clonal complexes, demographic data, antibiotic resistance, etc.). For all these ReportTree analyses, only samples that displayed a minimum number of loci called of 90% were used.

In parallel, for each PT isolate, the in silico identification of antibiotic resistance determinants was also performed using the PubMLST.org/neisseria database (Jolley et al. 2018) and then compared with antimicrobial susceptibility testing results whenever these data were produced.

### Statistical analysis

Simple descriptive statistics (absolute and relative frequencies) were used considering the date of onset for the temporal analysis of cases. Binomial 95% confidence intervals were calculated using SPSS version 28’s proportion confidence intervals. Incidence rates were calculated as the total number of IMD cases identified by the NRL in a given year (period from 2003 to 2020), using as denominator the Portuguese population from the PORDATA Portugal and European statistics censuses (PORDATA 2021). The Fisher-Freeman-Halton exact test was used to test the statistical significance difference in lethality between serogroups.

## Results

### Incidence rate and serogroup distribution

**Fig. 2:**
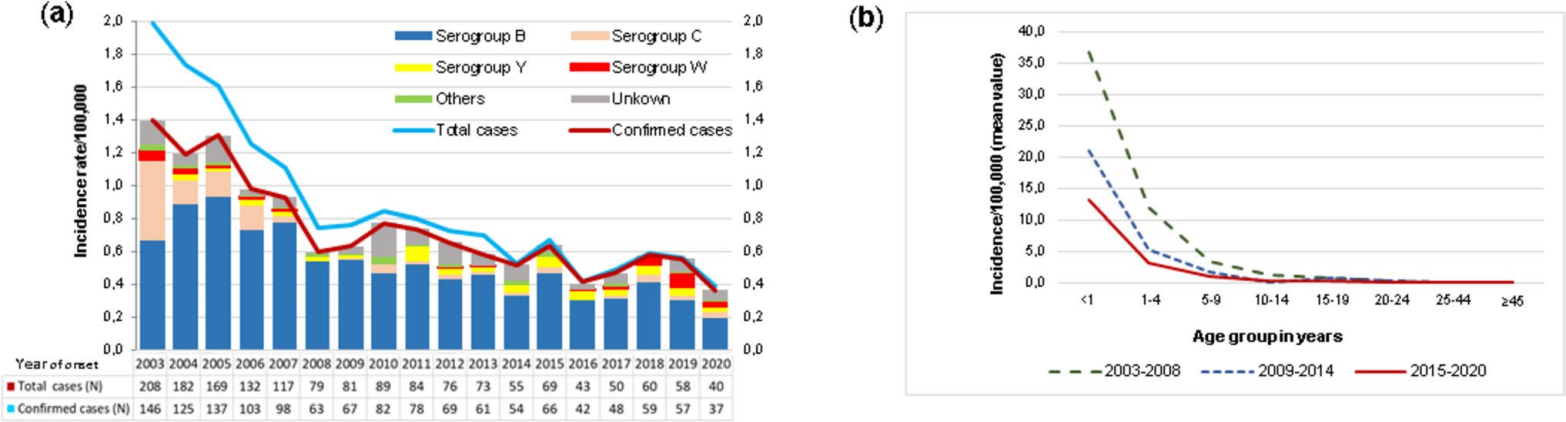
Invasive meningococcal disease incidence rates in Portugal from 2003 to 2020. **a** Incidence rate of IMD *per* year of onset and *per* serogroup. The total number of cases has been overlapping the number of confirmed cases over time. “Others” refers to serogroup A, X, E, Z, and capsule null cases. **b** Average incidence rate of IMD in three successive periods corresponding to three different measures implemented for IMD controlling

During the period 2003 to 2020, 1665 cases of IMD were reported in Portugal, with an average annual incidence rate of 0.89 cases *per* 100,000 population (Fig. 2a). Overall, a decrease in the annual incidence rate was observed, ranging from 1.99 (2003) to 0.39 (2020). Of the total number of IMD cases notified, 83.6% corresponded to confirmed cases (according to case definitions for reporting communicable diseases from the European Commission, 2018), and varied from 146 cases in 2003 to only 37 cases in 2020 (as a consequence of the implementation of public health measures during the COVID-19 pandemic), with the highest downward trend over time seen until 2008 (Fig. 2a; Supplementary Table 1).

Throughout the studied period, strains belonging to serogroup B were the most frequent ones [69.7% (970/1392); 95% CI: 67.2–72.1%], representing more than half of the IMD-confirmed cases *per* year (~ threefold in average), with exception of 2003. Regarding age distribution, 64.6% (627/970) of cases occurred in children up to 4 years of age. Regional variation of the annual incidence rates of MenB disease was observed, ranging from 0.13 to 0.55 *per* 100,000 population in the period 2012 to 2020 (Supplementary Fig. 1). The region of Baixo Alentejo presented the highest annual incidence rate estimate of MenB disease (0.55/100,000; 95% CI: 0.23–5.12), followed by the regions of Douro (0.51/100,000; 95% CI: 0.25–4.22), Azores (0.46/100,000; 95% CI: 0.24–3.67), Coimbra, and Beiras Serra da Estrela (0.45/100,000; 95% CI: 0.27–3.10).

Cases due to serogroup C represented 9.7% (135/1392; 95% CI: 8.2–11.3%) of all confirmed cases (Fig. 2a; Supplementary Table 1). This serogroup was the second most frequent until the end of 2006 (*n* = 97), becoming thereafter a rare cause of IMD restricted to adults and/or non-resident patients (*n* = 34). In 2010, one vaccine failure was reported in an 8-year-old child. Additionally, in 2011 and 2014, two cases were reported in unvaccinated children aged 11 years and 5 months, respectively, the latter not eligible for vaccination according to the National Immunization Program (NIP) in Portugal at that time (single dose at 12 months of age).

Serogroup Y accounted for 5.2% (72/1392; 95% CI: 4.1–6.4%) of all confirmed IMD cases occurring in all age groups (Fig. 2a; Supplementary Table 1). Despite no MenY was reported in 2003 and 2010, the number of MenY cases *per* year was small and homogeneous throughout the study period, except for 2011, where a peak of MenY cases was seen. The average incidence rate ranged from 0.48 cases to 0.22 cases *per* 100,000 children under the age of 12 months and 1–4 years of age, respectively. In the last 9 years, the annual incidence rate of MenY disease ranged between 0.03 and 0.17 *per* 100,000 population at a regional level, with the highest incidence observed in Coimbra (0.17 *per* 100,000 population), followed by Douro and Cávado (0.11 *per* 100,000 population).

Thirty-six cases due to serogroup W (2.6%; 95% CI: 2.6–3.5%) were identified over the study period (Fig. 2a; Supplementary Table 1). From 2003 to 2005, 12 cases of MenW were registered, 75% of which affecting children under 4 years of age, followed by an 11-year period with only five cases. From 2017 to 2019, there was an increasing number of cases (*n* = 19), initially affecting adults aged over 45 years but in 2019, MenW cases were mostly seen in children up to 4 years of age with an average incidence rate of 3.47 cases to 0.87 cases *per* 100,000 children aged under 1 year and 1–4 years old, respectively. From 2012 to 2020, the annual incidence of MenW disease ranged from 0.02 to 0.12 *per* 100,000 population by region, with Alto Tâmega presenting the highest annual incidence (0.12 *per* 100,000 population), followed by Coimbra (0.10 *per* 100,000 population). No cases of MenW IMD have been reported in the Azores archipelago.

Finally, 2.2% of all IMD-confirmed cases consisted of non-groupable (*n* = 23) and capsule null (*n* = 3) strains, as well as of cases due to serogroups A, X, E, and Z (each with one isolate) (Fig. 2a; Supplementary Table 1). Moreover, 149 invasive strains were cataloged as “unknown serogroup” since they have not been sent to the reference laboratory.

Given the different measures adopted to control IMD over the studied 18-year period, a comparative analysis of incidence rate by age group was performed *per* consecutive 6-year intervals (Fig. 2b). The highest incidence rate was observed in children under 12 months (mean 24.70/100,000; 95% CI: 22.5–27.2), markedly decreasing in the 1–4 years age group (mean 7.3/100,000; 95% CI: 6.7–7.9). For both age groups, the highest rates were seen within the 2003–2008 interval, followed by the 2009–2014 period, and to a lesser extent 2015–2020. This downward trend over time is not surprising, corroborating the timing of the introduction of MCC (2006–2007) and 4CMenB (2014) vaccines as IMD control measures. The age groups above 10–14 years showed very low incidence rates for all studied intervals.

### Case fatality rate

From 2003 to 2020, 118 deaths due to IMD were reported, 86 from confirmed cases and 32 from possible/probable cases. The average case fatality rate (CFR) registered in this period was 7.1%, ranging from 2.2% in 2010 to 10.6% in 2003 (data not shown). Analysis by age group revealed that the lowest CFR was recorded in the 15–19 age group (3.8%), reaching a high value of 13.6% in individuals aged 45 years or more. For children aged less than 12 months, the average CFR was 6.6%. Serogroup B was responsible for most deaths. However, the lethality of MenB (5.4%) was the lowest when compared to the lethality of the other serogroups, which varied between 8.3% (MenW) and 9.7 (MenY), with no statistical difference in the lethality of MenB (*p* = 0.228).

### Genetic diversity of Portuguese invasive *N. meningitidis* isolates

**Fig. 3:**
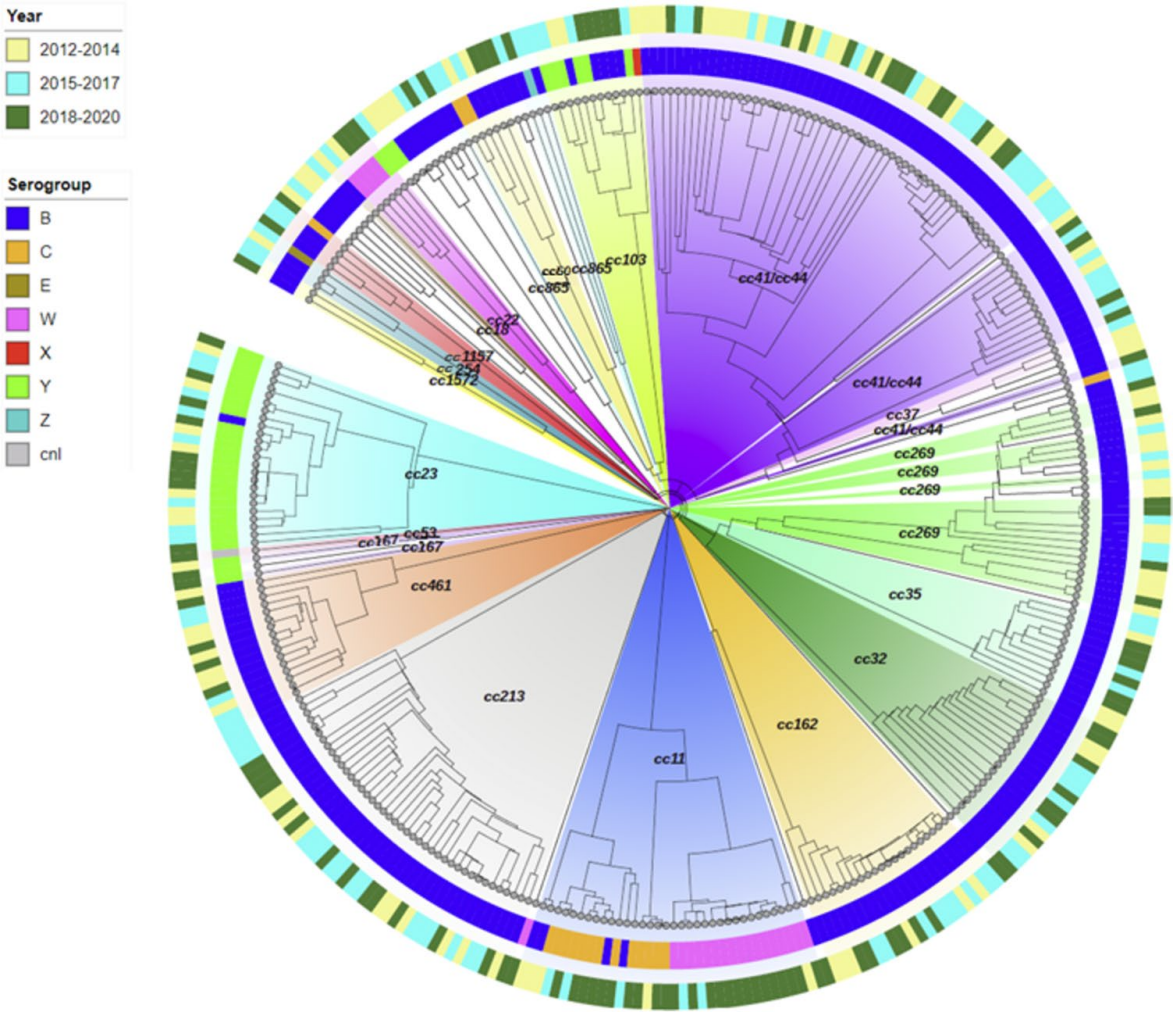
Hierarchical clustering tree showing the genetic relationship of all invasive *N. meningitidis* strains identified by the Portuguese National Reference Laboratory during the period 2012 to 2020. The genetic diversity among isolates was evaluated by a gene-by-gene analysis using the newly improved cgMLST schema v2, with 1422 *N. meningitidis* core-*loci* (Jolley et al. 2018). Phylogenetic tree was constructed using the hierarchical single-linkage method, based on the number of shared cgMLST alleles among 327 validated isolates (two isolates were discarded since displayed < 90% loci-called) and was drawn using microreact (https://microreact.org/). Isolates are depicted by small black circles that are differentially colored, the external ring according to their year of isolation and the internal ring according to their serogroup. The internal shading displayed in several colors shows the partitions of the various clonal complexes

From 2012 to 2020, 329 (95.4%) out of 345 invasive isolates from confirmed cases were characterized based on WGS. Phylogenetic analysis encompassing all validated *N. meningitidis* isolates from the PT (two isolates were discarded as they displayed < 90% loci-called; see “Methods” for details) (Fig. 3) confirmed the segregation of isolates into 20 clonal complexes (cc), some of them associated with multiple serogroups. That was the case of the cc11 (MenB, MenC, and MenW), cc23 (MenY and MenB), and cc103 (MenB and MenY).

**Fig. 4:**
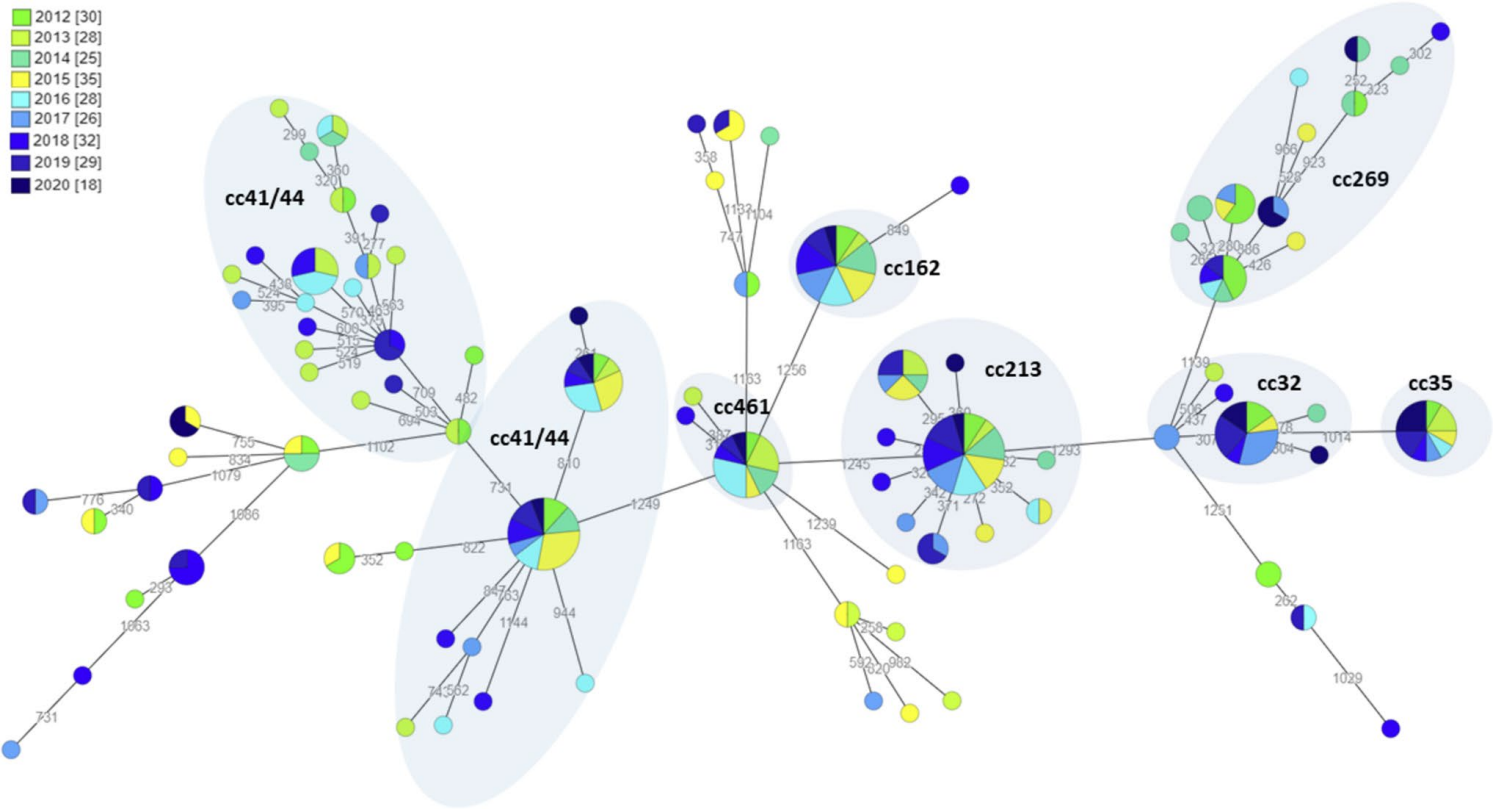
Genetic clusters identified among invasive Portuguese isolates of *N. meningitidis* serogroup B during the period 2012 to 2020. The genetic diversity among isolates was evaluated by a gene-by-gene analysis using the cgMLST schema v2, with 1422 *N. meningitidis* core-*loci*. The Minimum spanning tree was generated with the MSTreeV2 method of GrapeTree and was based on allelic diversity found among 251 validated MenB isolates (Mixão et al. 2023). For a better visualization of the identified gene clusters, nodes were collapsed. Filled small circles (whose size is proportional to the number of isolates it represents) represent unique allelic profiles and were colored by year of isolation with clonal complexes represented by large, shaded circles. The numbers in grey on the connecting lines represent the allele differences between isolates

The highest genetic diversity was observed for MenB, which represented 76.7% (251/327) of all validated sequenced isolates in this period. Indeed, MenB isolates were found to fall into 17 different clonal complexes, with cc41/44 (*n* = 66, [26.3%]) being the most prevalent one, followed by cc213 (*n* = 41, [16.3%]), and to a lesser extent cc162 (*n* = 21, [8.4%]), cc269 (*n* = 20, [7.9%]), and cc32 (*n* = 19, [7.6%]) (Fig. 3). Among cc41/44, MenB isolates were mostly from subtype P1.7–2,4 (27.3%), followed by both P1.22,14–6 and P1.18–1,34 subtypes (15.2% each) (Supplementary Table 2). On the other hand, P1.22,14 (92.7%) and P1.7–2,4 (90.5%) subtypes were predominant among MenB isolates assigned to the cc213 and cc162, respectively, while subtype P1.22,9 (60.0%) was mostly seen in MenB cc269 isolates. Isolates with unassigned clonal complex corresponded to 11.2% (*n* = 28) of all sequenced MenB. Although most clonal complexes were recurrently identified over time (Fig. 4), a slight decreasing trend was observed in the number of MenB cc41/44 cases, while the inverse was seen for MenB cc213. Of note, 73.7% of MenB cc32 cases (*n* = 14) were identified in the last 4 years of the study period, affecting mostly children up to 4 years old, followed by adults aged over 45 years. Moreover, several genetic clusters enrolling isolates from multiple years were identified within some clonal complexes, suggesting the predominance of more fitted MenB clones of specific subtypes. This was clearly visible for cc41/44, cc461, and cc162, for instance, where genetically related MenB isolated over the years were segregated together (Fig. 4). In general, with the exception of cc213 (not reported in 12 regions), the most prevalent clonal complexes were identified in almost all Portuguese regions (Supplementary Table 2). However, this observation should be viewed with caution in view of the low number of cases reported in each region. Finally, comparative genome analysis revealed that five MenB isolates underwent capsular switching. Indeed, one isolate assigned to cc23 evidenced a capsular switching from Y to B, and four isolates assigned to cc11 (B:P1.5–1,10–8:F3-6:cc11) underwent a capsular switching from C to B (Fig. 3).

Concerning MenY, the identified 35 isolates represented 11.0% of all validated sequenced isolates and were assigned into four clonal complexes (Fig. 3). The cc23 was the most frequently observed among MenY representing 65.7% of all MenY isolates (*n* = 23, with 42.9% from subtype P1.5–2.10–1), being consistently reported in multiple geographical regions over the years (Supplementary Table 2). The remaining isolates were assigned to cc103 (*n* = 7, with 71.4% from subtype P1.5–1.10–46), cc167 (*n* = 2), cc22 (*n* = 1), and two unassigned.

Regarding MenW, the core-genome phylogenetic analysis pointed to a clonal character of the 22 isolates (6.7%), identified since 2012 with a predominance of cc11 subtype P1.5,2 (*n* = 17, [77.3%]), and to a lesser extend of cc22 subtype P1.18–1,3 (*n* = 4, [18.2%]) (Fig. 3, Supplementary Table 2). One isolate ST-9316 was unassigned to any clonal complex. While no visible trend on circulation of MenW cc22 was found throughout time, 94.1% of MenW cc11 isolates were seen circulating from 2017 onwards.

Of the total 17 MenC isolates identified from 2012 to 2020, most belonged to cc11 (*n* = 13, [76.5%]) with 61.5% from subtype P1.5–1.10–8, and to a lesser extend subtype P1.5.2 (30.8%). Interestingly, despite the clear predominance of this clonal complex since 2012, MenC cc11 isolates were found to be rare during the epidemic period up to 2006, in which the already extinct MenC cc8 was dominant (reference laboratory data).

Other serogroups responsible for IMD were also found from 2012 onwards and included one MenX [X:P1.7–2.4:F3-9:ST-11209(cc103)] identified in 2014, one MenE [E:P1.5–1.2–2:F3-6:ST-2151(cc254)] from 2015, and one MenZ [Z:P1.7–1.1:F1-6:ST-14880(cc865)] from 2017. In 2020, a fatal case was identified due to an invasive capsule null isolate [cnl:P1.17–1.23–3:F1-5:ST-53(cc53)] in a patient who developed sepsis secondary to community-acquired pneumonia.

### Antimicrobial susceptibility

Antimicrobial susceptibility testing was performed on 296 (85.8%) out of the 345 invasive isolates from confirmed cases, identified from 2012 to 2020. The studied isolates were mostly MenB (*n* = 220), followed by MenY (*n* = 31), MenW (*n* = 22), and MenC (*n* = 18).

**Fig. 5:**
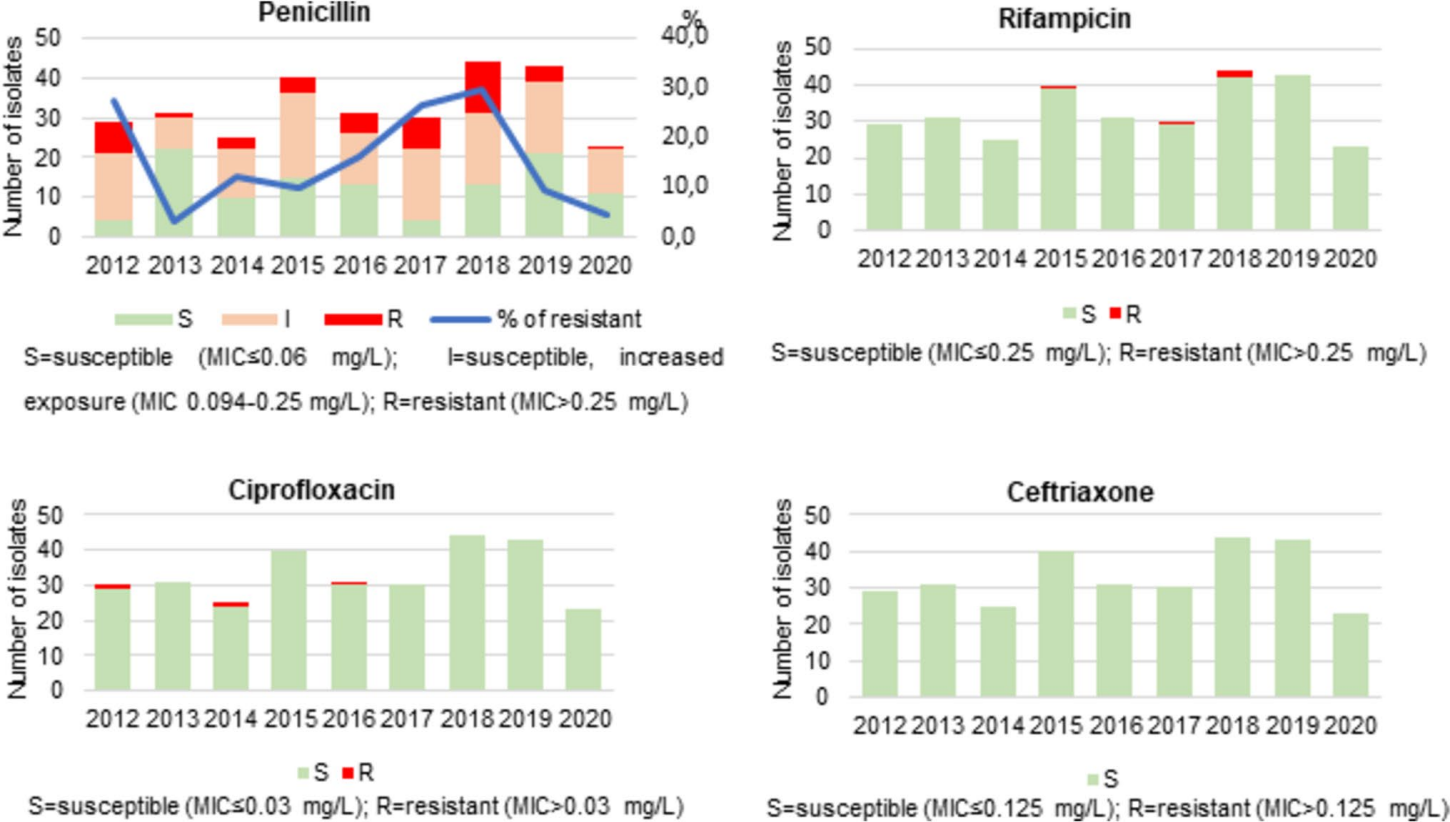
Distribution of antibiotic susceptibility to penicillin, rifampicin, ciprofloxacin, and ceftriaxone of invasive *N. meningitidis* isolates identified during the period 2012 to 2020 in Portugal. Antimicrobial susceptibility results were interpreted according to the European Committee for Antimicrobial Susceptibility Testing guidelines (EUCAST 2020)

Susceptibility (S) to penicillin was observed in 38.2% (*n* = 113) of the studied isolates, while reduced susceptibility to penicillin (susceptible, increased exposure) was observed in 45.9% (*n* = 136) (Fig. 5). Penicillin resistance (R) was noted for 15.9% of isolates (*n* = 47), ranging from 3.2% in 2013 to 29.5% in 2018. None of the isolates was β-lactamase producer. Despite year-to-year, variation in the number of penicillin-nonsusceptible isolates occurred no significant trends were detected. The largest proportion of both resistance and reduced susceptibly to penicillin was observed in MenC isolates (*n* = 14, [77.8%]), all but one belonging to cc11, followed by MenB (*n* = 139, [63.2%]) with a similar distribution by clonal complex, and MenY (*n* = 19, [61.3%]). The only MenZ isolate and a capsule null isolate (identified in 2017 and 2020, respectively) were found to be resistant to penicillin.

In a total of 293 isolates whole genome sequenced, we found a concordance of 75.1% (220/293) between phenotypic and genotypic antimicrobial susceptibility results. The highest discrepancies were observed in isolates characterized as susceptible (12.3%, 14/114) and reduced susceptibility (59.7%, 80/134) to penicillin based on phenotypic tests, with mutations in the gene encoding the penicillin-binding protein 2.

In general, 66.5% of all isolates non-susceptible to penicillin displayed the five well-characterized non-synonymous alterations (F504L, A510V, I515V, H541N, and I566V) in the gene encoding the penicillin-binding protein 2 (*penA*), with the *penA19* (17.6%), *penA9* (13.4%), and *penA14* (7.0%) alleles being the most frequent (Taha et al. 2007).

Four MenB isolates (*n* = 4, [1.4%]) with rifampicin resistance were identified between 2015 and 2018 (Fig. 5). One isolate was identified in 2015 with MIC > 32 mg/L due to a known nonsynonymous mutation (H552Y) in the *rpoB* gene (allele *rpoB65)* (Lodi et al. 2020). From 2017 to 2018, three isolates with MIC values of 0.38 mg/L (in 2017, allele *rpoB18*), 1.02 mg/L (in 2018, allele *rpoB28*), and > 32 mg/L (in 2018, allele *rpoB288*) were accounted, all also penicillin-nonsusceptible. The remaining isolates were sensitive to rifampicin with MIC values ≤ 0.25 mg/L.

Regarding ciprofloxacin, almost all the studied isolates (99.3%) were phenotypically susceptible (Fig. 5). The exception was three MenB sporadically isolated from children under 5 years old in 2012, 2014, and 2016, which exhibited low-level ciprofloxacin resistance (MIC values ranging from 0.094 to 0.38 mg/L). However, none of these isolates exhibited the typical mutations commonly associated with ciprofloxacin resistance in the gyrase gene (*gyrA*) (Shultz et al. 2005). These ciprofloxacin-resistant isolates also showed reduced susceptibility or resistance to penicillin.

All tested isolates were susceptible to ceftriaxone with MIC values ranging from ≤ 0.002 mg/L (59.7%) to 0.047 mg/L (0.5%).

## Discussion

This study provides a detailed description of the epidemiology of IMD in Portugal, based on epidemiological data and molecular characterization of *N. meningitidis* strains over 18 years of laboratory-based surveillance.

Our results showed a decreasing trend of the IMD incidence rate from 2003 to 2020, with an average mortality rate of 7.1%, both in line with data from most of the European countries notifying TESSy during the same period (ECDC 2022b). In general, serogroup B strains were the major cause of IMD, followed by MenC and MenY. However, an increased number of IMD cases caused by serogroup W was observed from 2017 onwards, initially in adults and later in children under 4 years of age. The highest downward trend in IMD incidence over time was seen up to 2008. This was mostly due to the decrease of MenC cases, as a consequence of the inclusion of the MenC vaccine in the national immunization plan in 2006, and to the successful catch-up campaign in the population under 19 years of age (the estimated age of vaccination coverage ranged between 88 and 98%) (DGS 2005, 2012). Nevertheless, we had previously observed a decrease in the serum bactericidal antibody levels against MenC after childhood immunization likewise reported by other countries (Gonçalves et al. 2015; Borrow et al. 2013; Ishola et al. 2012). Despite this finding leading several countries to consequently introduce a booster dose in adolescence, Portugal has not changed its vaccination policy, which highlights the need for close monitoring of this serogroup. On the other hand, a relatively stable decrease in the incidence rate of MenB disease was also observed since 2008, slightly more heightened after 2015, mainly in children under 4 years of age. We believe that the marketing of the 4CMenB vaccine in Portugal in 2014 could be a plausible reason for this event, given the progressive increase in the immunization status of the Portuguese population that occurred in the years 2015 to 2019 (Bettencourt et al. 2022a). Despite this, we observed higher incidence rates of MenB disease in three regions of the country that are characterized by an elderly population, namely Serra da Estrela, Douro, and Baixo Alentejo. Although in a pure speculative basis, we hypothesize that this finding may be due to several causes, such as the lack of vaccination, climatic reasons (as these are among the coolest regions during the winter), or associated with genetic factors that contribute to host susceptibility and the development of IMD (Kinlin et al. 2009; Paireau et al. 2016; Davila et al. 2010).

Phylogenetic analysis showed that the meningococcal population was highly diverse, with most clonal complexes recurrently identified over time. The MenB population was the most diverse, grouping into several clonal complexes, being the cc41/44 and cc213 the most prevalent, mirroring the scenario of other European countries (ECDC 2022a). However, we must remain vigilant regarding the increasing trend observed in some clonal complexes, like cc213 and cc32, for instance. While the former is of particular interest considering the predicted low coverage of these strains by the 4CMenB vaccine, cc32 displays an epidemic potential, already causing several large outbreaks in Norway and France (Bettencourt et al. 2022a; Smith et al. 2006; Levy et al. 2012). Concerning MenY, a predominant association with cc23 was seen affecting all age groups, a trend that follows the emergence of cc23 strains in several European countries since the early 2000s (Bröker et al. 2014). Genomic characterization of MenW revealed a shift among circulating strains during the last 5 years, with cc11 predominating since 2017. We previously showed that the increase in Portuguese MenW cc11 cases was related to the emergence of the “Original UK” and “UK 2013” strains in the UK, likewise, seen in many European countries since 2014 (Bettencourt et al. 2022b; Ladhani et al. 2015; Knol et al. 2017; Eriksson et al. 2018). Since both “Original UK” and “UK 2013” strains are responsible for IMD with unusual clinical presentation and seem to be associated with higher fatality rates, some countries, such as the UK in 2015, and more recently the Netherlands and Spain, have introduced the tetravalent MenACWY vaccine into their national immunization plans in order to reduce IMD by MenW (Campbell et al. 2015; Knol et al. 2018; Álvarez García et al. 2021). We observed a heterogeneous temporal distribution for MenW and MenC. For example, 94.1% of MenW cc11 isolates were identified in the 2017–2020 period. Also, the epidemic strains MenC cc8 have not been identified since 2006, when the vaccine was introduced in the NIP, and MenC cc11 that was previously rare, became the most prevalent until 2020 (Simões 2012).

Albeit relatively rare, a few capsular switching events were found throughout the study period. Based on our phylogenetic analysis, most of these events are associated with the hypervirulent cc11 and enrolled isolates with a genome backbone of serogroup C, while presenting a capsule synthesis region derived from a MenB strain. In Portugal, whereas the 4CMenB vaccine covers MenC and MenW cc11 strains, the MenB cc11 strains are not covered (Bettencourt et al. 2022a). Considering that capsular switching may lead to the emergence of strains with high pathogenic potential together with the fact that cc11 is responsible for large outbreaks of serogroups C and W, genomic surveillance should not be neglected (Lancellotti et al. 2006; Castilla et al. 2009; Stefanelli et al. 2019; Lucidarme et al. 2017, 2015; Mustapha et al. 2015).

All invasive PT isolates showed susceptibility to ceftriaxone (third-generation cephalosporin), which is the empirical treatment of choice for *N. meningitidis* infections (Prata et al. 2013). To our knowledge, no cases of resistant *N. meningitidis* strains have been reported so far to this drug (Deghmane et al. 2017, 2023). However, most isolates were found to display reduced susceptibility or even resistance to penicillin throughout the studied period, affecting patients from all ages (Fig. 3). This high level of non-susceptibility to penicillin within the Portuguese meningococcal population can be explained by the successful expansion of these strains with altered *penA* alleles (Taha et al. 2007; Deghmane et al. 2023). On the other hand, resistance to rifampicin and ciprofloxacin, which are two antibiotics commonly used for chemoprophylaxis of close contacts of IMD patients, was rare, occurring solely as sporadic MenB cases in specific years. Noteworthy, all isolates identified as resistant to these clinically relevant antibiotics were also non-susceptible to penicillin. All these findings are in line with antimicrobial resistance surveys previously conducted by several countries, like France, Italy, UK, Brazil, or USA (Deghmane et al. 2017; Vacca et al. 2018; Willerton et al. 2021; Gorla et al. 2018; Potts et al. 2022). Despite the penicillin susceptibility surveillance is usually performed, this antibiotic is not used as a first-line prophylactic drug for *N. meningitidis* infection in Portugal. Considering that the observed dual resistance represents a risk to infection control, the emergence and spread of non-susceptible invasive isolates must be monitored at both national and global levels to ensure their continued effective use or the development of new treatment options.

Globally, the results of this study underline the need of continuous surveillance of *N. meningitidis* infections at both phenotypic and genomic levels*.* In addition, it highlighted genome-based surveillance as an important tool for identifying the emergency of novel virulent strains, and ultimately for the understanding of the evolutionary dynamics of this species in Portugal and subsequent control of IMD.

### Supplementary Information

Below is the link to the electronic supplementary material.

Supplementary file1 (PDF 109 KB)

Supplementary file2 (XLSX 12 KB)

Supplementary file3 (XLSX 36 KB)

Supplementary file4 (PDF 131 KB)

## Data Availability

Raw sequence reads used in the present study were deposited in the European Nucleotide Archive (ENA) under the study accession number PRJEB36474. Isolate metadata and genome sequence assemblies are available from the PubMLST Neisseria repository (http://pubmlst.org/neisseria/).
