# Supplementary material for: Epidemiology and genetic diversity of invasive Neisseria meningitidis strains circulating in Portugal from 2003 to 2020
Source: Int Microbiol. 2023 Dec 7;27(4):1125–36. doi: 10.1007/s10123-023-00463-w (PMC11300501; doi:10.1007/s10123-023-00463-w)
Supplement: Supplementary file 1 — Supplementary file1 (PDF 109 KB) [file 10123_2023_463_MOESM1_ESM.pdf]

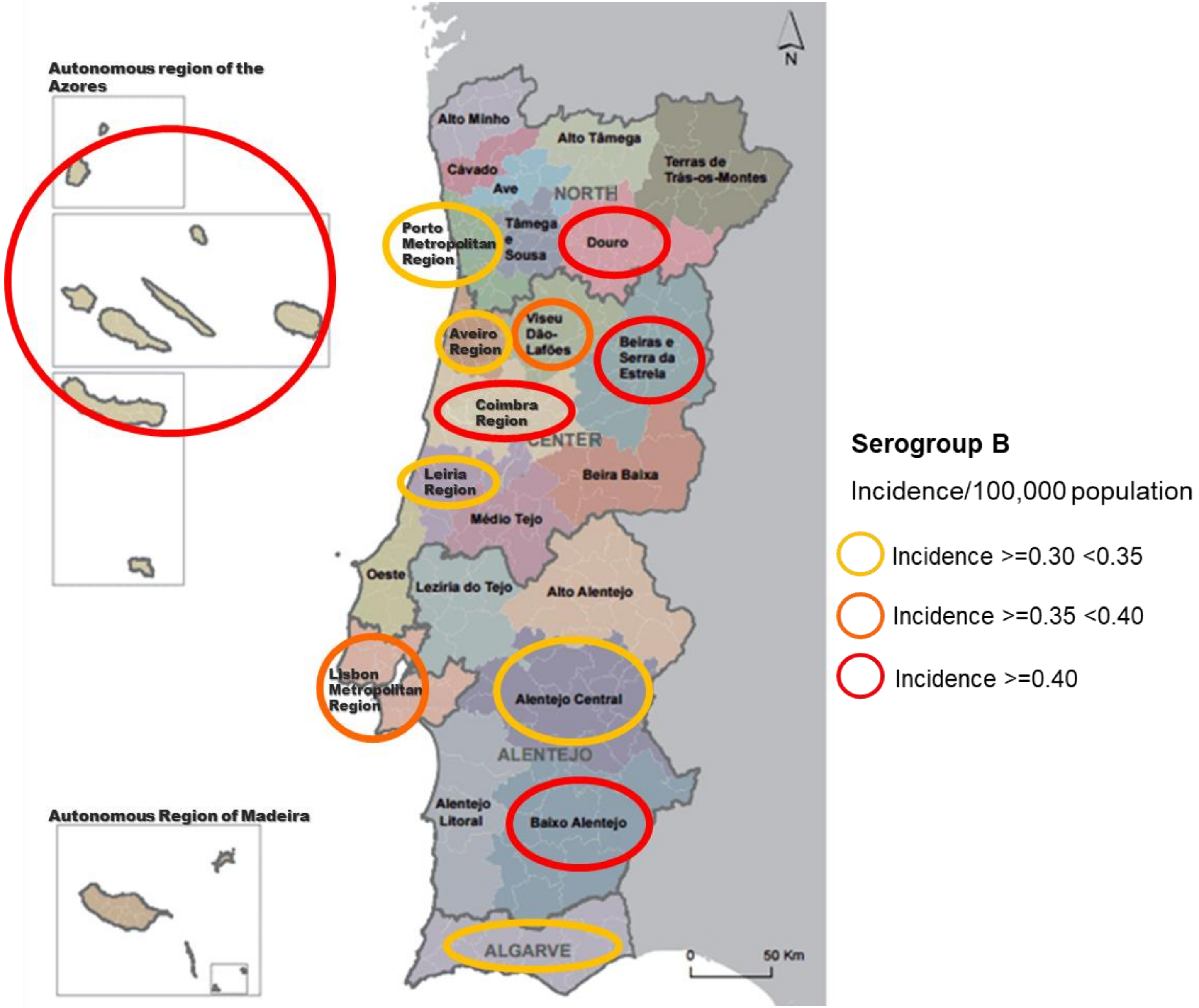

Supplementary Fig. 1 Map of Portugal with the incidence of serogroup B invasive meningococcal disease in the period 2012-2020 by geographical region. Geographical regions were classified according to the Nomenclature of Territorial Units for Statistics (NUTS III). The curves are presented in three colours: red for regions of high incidence (Incidence above the 85 percentile); orange for intermediate incidence (Incidence between percentiles 76 and 85), and yellow for low incidence (Incidence between percentiles 50 and 75)
