# Supplementary material for: Epidemiology and genetic diversity of invasive Neisseria meningitidis strains circulating in Portugal from 2003 to 2020
Source: Int Microbiol. 2023 Dec 7;27(4):1125–36. doi: 10.1007/s10123-023-00463-w (PMC11300501; doi:10.1007/s10123-023-00463-w)
Supplement: Supplementary file 4 — Supplementary file4 (PDF 131 KB) [file 10123_2023_463_MOESM4_ESM.pdf]

VigLab_DIM_Network Group

Cristina Toscano[6], Elsa Gonçalves[6], José Melo Cristino[7], Maria Dinah Carvalho[7], Alberta Faustino[8], Aurélio Mesquita[8], Luísa M. Vieira[9], Claúdia MAC. Branco[9], Maria Favila Menezes[10], Maria José Rego de Sousa[10], José Diogo[11], Fátima Vale[12], Rita Gralha[12], Valquíria Alves[13], Antónia Read[13], Fátima Silva[14], Adriana Pedrosa[14], Isabel Vale[15], Maria João Tomás[15], Paulo Martinho[16], Gema Mira[16], Paula Mota[17], Hermínia Marques[17], Ezequiel Moreira[18], Carla Ferreira[18], Gabriela Abreu[19], Aurélia Selaru[19], Patrícia Amantegui Ibarzabal[20], Paula Cristina J. Gouveia Pestana[20], Sandra Vieira[21], Mota Freitas[21], Margarida Feijó Pinto[22], [23]Ana Filipa Resende, [23]Andreia Sofia Bernardo, Rui Ferreira[24], Henrique Oliveira[25], Fernando Rodrigues[25], Ana Filipa M. Vicente[26], Nuno Canhoto[26], Manuela Ribeiro[27], Angélica Ramos[27], Gina Marrão[28], [29]Maria Calle Velles, [29]Mariana Viana, [30]Adriana Coutinho, [30]Elsa Lopes

[6] Serviço de Patologia Clínica, Centro Hospitalar de Lisboa Ocidental, EPE

[7]Serviço de Patologia Clínica, Centro Hospitalar Universitário Lisboa Norte, EPE

[8] Serviço de Patologia Clínica, Hospital de Braga, EPE

[9]Unidade de Genética e Patologias Moleculares, Hospital Divino Espírito Santo de Ponta Delgada, EPE

[10]Serviço de Patologia Clínica, Centro de Medicina Laboratorial Germano de Sousa, Hospital CUF Descobertas

[11]Serviço de Patologia Clínica, Hospital Garcia de Orta, EPE

[12]Serviço de Patologia Clínica Unidade Local de Saúde da Guarda, EPE

[13]Serviço de Patologia Clínica, Unidade Local de Saúde de Matosinhos, EPE

[14]Serviço de Patologia Clínica, Centro Hospitalar de Entre o Douro e Vouga, EPE, Hospital de São Sebastião

[15]Serviço de Patologia Clínica, Centro Hospitalar Tondela Viseu, EPE

[16]Serviço de Patologia Clínica, Unidade Local de Saúde do Norte Alentejano, EPE, Hospital Santa Luzia de Elvas

[17]Serviço de Patologia Clínica, Hospital da Senhora de Oliveira – Guimarães, EPE

[18]Serviço de Patologia Clínica, Centro Hospitalar do Médio Ave, EPE

[19]Serviço de Patologia Clínica, Centro Hospitalar de Vila Nova de Gaia/Espinho, EPE

[20]Serviço de Patologia Clínica, Centro Hospitalar Cova da Beiram EPE, Hospital Pêro da Covilhã

[21]Serviço de Patologia Clínica, Unidade Local de Saúde do Alto Minho, EPE, Hospital de Santa Luzia

[22]Serviço de Patologia Clínica, Centro Hospitalar Universitário Lisboa Central, EPE

[23]Serviço de Patologia Clínica, Centro Hospitalar de Setúbal, EPE

[24]Serviço de Patologia Clínica, Centro Hospitalar Universitário do Algarve, EPE, Hospital de Faro.

[25]Serviço de Patologia Clínica, Centro Hospitalar e Universitário de Coimbra, EPE

[26]Serviço de Patologia Clínica - Serviço de Saúde da Região Autónoma da Madeira (SESARAM), Hospital Dr. Nélio Mendonça

[27]Serviço de Patologia Clínica, Centro Hospitalar Universitário de São João, EPE

[28]Serviço de Patologia Clínica, Centro Hospitalar de Leiria, EPE

[29]Serviço de Patologia Clínica, Centro Hospitalar do Tâmega e Sousa, EPE

[30] Serviço de Patologia Clínica, Hospital do Espírito Santo de Évora, EPE
